# Developmental Role and Regulation of *cortex*, a Meiosis-Specific Anaphase-Promoting Complex/Cyclosome Activator 

**DOI:** 10.1371/journal.pgen.0030202

**Published:** 2007-11-16

**Authors:** Jillian A Pesin, Terry L Orr-Weaver

**Affiliations:** 1 Department of Biology, Massachusetts Institute of Technology, Cambridge, Massachusetts, United States of America; 2 Whitehead Institute, Cambridge, Massachusetts, United States of America; The University of North Carolina at Chapel Hill, United States of America

## Abstract

During oogenesis in metazoans, the meiotic divisions must be coordinated with development of the oocyte to ensure successful fertilization and subsequent embryogenesis. The ways in which the mitotic machinery is specialized for meiosis are not fully understood. *cortex*, which encodes a putative female meiosis-specific anaphase-promoting complex/cyclosome (APC/C) activator, is required for proper meiosis in *Drosophila*. We demonstrate that CORT physically associates with core subunits of the APC/C in ovaries. APC/C^CORT^ targets Cyclin A for degradation prior to the metaphase I arrest, while Cyclins B and B3 are not targeted until after egg activation. We investigate the regulation of CORT and find that CORT protein is specifically expressed during the meiotic divisions in the oocyte. Polyadenylation of *cort* mRNA is correlated with appearance of CORT protein at oocyte maturation, while deadenylation of *cort* mRNA occurs in the early embryo. CORT protein is targeted for degradation by the APC/C following egg activation, and this degradation is dependent on an intact D-box in the C terminus of CORT. Our studies reveal the mechanism for developmental regulation of an APC/C activator and suggest it is one strategy for control of the female meiotic cell cycle in a multicellular organism.

## Author Summary

Meiosis is a modified cell cycle that generates four gametes, each containing half the genetic content of the parent cell, through a reductional division followed by an equational division without an intervening DNA synthesis phase. During oogenesis of multicellular organisms, proper coordination of the meiotic divisions with the development of the oocyte is crucial for successful fertilization and the initiation of zygotic development. Very little is known about how general cell-cycle regulators as well as meiosis-specific regulators contribute to this coordination. In this study we describe the role and developmental regulation of *cortex*, a meiosis-specific activator of the anaphase-promoting complex/cyclosome (APC/C). CORT protein physically associates with the APC/C and triggers the sequential degradation of mitotic cyclins in meiosis. We find that *cortex* is subject to both post-transcriptional and post-translational regulatory mechanisms, which result in expression of CORT protein being restricted to the meiotic divisions. This developmental regulation may be important for proper meiosis as well as the transition from the completion of meiosis to mitotic divisions in the early embryo.

## Introduction

Developmental regulation of meiosis is crucial for generating viable eggs and sperm and, thus, a successful fertilization event. Meiosis is a modified cell cycle in which segregation of homologous chromosomes is followed by segregation of sister chromatids without an intervening S phase. These unique divisions are controlled by general mitotic cell-cycle regulators as well as specialized meiotic proteins [1]. During oogenesis in multicellular organisms, meiosis presents a particular regulatory challenge. The meiotic divisions must be coordinated tightly with growth and development of the oocyte to allow for oocyte differentiation and to ensure that the completion of meiosis is coordinated with fertilization. To achieve this coordination, oocytes arrest at prophase I and again at metaphase I or metaphase II and are released from these arrests through processes called oocyte maturation and activation, respectively [2,3]. Furthermore, additional specialized cell-cycle regulation is required for the transition between meiosis and restart of the cell cycle in embryogenesis. In *Drosophila*, meiosis is completed without cytokinesis in the same common cytoplasm in which the rapid mitotic divisions of embryogenesis begin. Upon fertilization, the oocyte must quickly inactivate meiotic regulators to prevent interference with embryonic mitotic cycles. The ways in which general mitotic proteins act together with meiosis-specific proteins to meet the multiple regulatory challenges of meiosis in metazoans are not well understood.

The anaphase-promoting complex/cyclosome (APC/C) plays a critical role in mitosis, but much remains to be understood about its function in meiosis. The APC/C is a large E3 ubiquitin ligase composed of at least 12 core subunits, which targets specific substrate proteins for degradation by the 26S proteasome [4]. In mitosis, the APC/C is crucial for proper cell division through targeting of key substrates. Securin, an inhibitor of separase, must be degraded to allow for cleavage of cohesin and subsequent segregation of sister chromatids, and mitotic cyclins must be degraded to allow for the metaphase to anaphase transition and events associated with mitotic exit [5–8]. In addition, the APC/C targets many other proteins for degradation including proteins involved in spindle function and regulators of DNA replication [9,10].

Substrate specificity is conferred to the APC/C by activator proteins Cdc20/Fizzy and Cdh1/Fizzy-related, which recognize substrate proteins containing D-box or KEN box motifs [11–15]. Regulation of these specificity factors is one crucial way by which APC/C activity is modulated. Cdc20 is transcribed and translated during S phase and G2, phosphorylated in mitosis, and degraded in an APC/C^Cdh1^-dependent manner in G1 [13,16–19]. Phosphorylation of several APC/C subunits in mitosis facilitates the ability of Cdc20 to bind to and activate the APC/C [18,20–23]. Levels of Cdh1 are constant in mitosis and lowered in late G1 and S, but inhibitory phosphorylation of Cdh1 prevents its association with APC/C during S, G2, and M phases [16,18,24]. Thus, differential regulation of Cdc20 and Cdh1 directs their transient association with the APC/C at different times during the cell cycle to target specific subsets of proteins for degradation.

In meiosis, the role of the APC/C and its regulation is less clear. An appealing hypothesis is that the meiotic divisions are driven in part by the degradation of specific meiotic APC/C substrates, and thus, the APC/C must require unique regulation during these divisions. In yeast, it is known that disjunction of homologous chromosomes in meiosis I and sister chromatids in meiosis II requires APC-mediated destruction of Pds1/securin to release separase for cleavage of cohesin [25–27]. In multicellular organisms, however, a requirement for the APC/C in meiosis has been more difficult to demonstrate. Mutations in or RNA interference against APC/C subunits in Caenorhabditis elegans result in a metaphase I arrest [28,29]. In *Drosophila*, mutations in *fzy* cause both meiosis I and meiosis II arrests [30]. Several studies in mouse oocytes have shown that APC/C-mediated protein degradation is required for homolog disjunction and polar body extrusion [31–34]. However, inhibiting APC/C subunits by depletion or antibody injections in Xenopus laevis does not prevent the metaphase I to anaphase I transition but does cause arrest in metaphase II [35,36]. The reasons behind this apparent inconsistency in *Xenopus* remain unknown.

One way in which the APC/C may be regulated uniquely in meiosis is through its association with meiosis-specific activators. Cdc20/FZY family members that are expressed exclusively in meiosis have been identified in yeast. In Saccharomyces cerevisiae, Ama1 activates the APC/C after meiosis I and is required for spore wall assembly [37–39]. Similarly, in S. pombe, *fzr1/mfr1* is required for proper spore formation after the completion of the meiotic divisions [40,41]. The identification and study of meiosis-specific APC/C activators are starting points from which to understand the unique regulation of the APC/C during meiosis as well as to identify meiosis-specific substrates of the APC/C.

*Drosophila* provides the best candidate for a meiosis-specific APC/C activator in metazoans. *cortex* (*cort*) encodes a distant member of the Cdc20/FZY family and is expressed exclusively in oogenesis and early embryogenesis [42]. *cort* is required for proper female meiosis. Eggs laid by *cort* mutant mothers display aberrant chromosome segregation in meiosis I and arrest terminally in metaphase II [43,44]. In addition, *cort* mutant eggs contain elevated levels of mitotic cyclins, and misexpression of *cort* causes a decrease in levels of mitotic cyclins [30]. *cort* presents a unique opportunity for understanding the function and the developmental regulation of the APC/C during meiosis in a multicellular organism. *Drosophila* is an ideal system for studying female meiosis because different meiotic stages can be distinguished easily by cytology and isolated by microdissection. In this study, we demonstrate that CORT interacts biochemically with the APC/C during female meiosis and reveal a mechanism for developmental regulation of *cort* through both post-transcriptional and post-translational processes.

## Results

### CORT Protein Is Associated with the APC/C In Vivo

A recent study suggested that the cortex gene encodes a functional activator of the APC/C [30]. This assertion was based on the ability of *cort* to affect cyclin protein levels. Levels of mitotic cyclins are elevated in *cort* mutants, and misexpression of *cort* causes decreased levels of cyclins in wing imaginal discs. While these data are strong evidence of *cort*'s function as an activator of the APC/C, demonstration of a physical association between CORT and the APC/C during oogenesis is lacking. We looked for an association between CORT and the APC/C by co-immunoprecipitation. We made transgenic lines with a MYC-tagged form of *cort* under control of the UAS response element and drove MYC-CORT expression in the female germline using *nos-gal4*. MYC-CORT is functional, because expression of this transgene rescued the meiotic arrest in progeny of *cort^RH65^* mutant females (Table S1).

**Figure 1:**
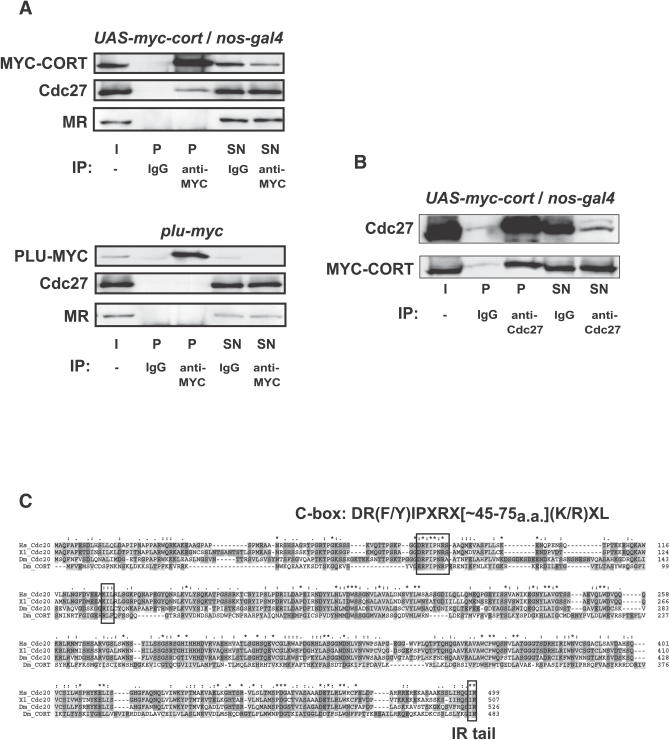
CORT Protein Associates with the APC/C In Vivo (A) Cdc27 co-immunoprecipitates with MYC-CORT. Top panel: MYC-CORT was immunoprecipitated from whole ovary extracts from *UAS-myc-cort*/*nos-gal4* females with anti-MYC or control mouse IgG, and immunoprecipitates were examined for the presence of APC/C subunits, Cdc27 and MR/APC2. Bottom panel: Unrelated MYC-tagged control PLU-MYC was immunoprecipitated from whole ovary extracts from *plu-myc* transgenic females with anti-MYC or control mouse IgG, and immunoprecipitates were examined for the presence of APC/C subunits. I, input; P, pellet; SN, supernatant. (B) MYC-CORT co-immunoprecipitates with Cdc27. Cdc27 was immunoprecipitated from whole ovaries from *UAS-myc-cort*/*nos-gal4* females with anti-Cdc27 or a control rabbit IgG, and immunoprecipitates were examined for the presence of MYC-CORT. (C) CORT contains motifs conserved in the Cdc20/FZY family of proteins. CORT protein sequence was hand aligned with Cdc20/FZY protein sequences from human (Hs), *Xenopus* (Xl), and *Drosophila* (Dm). The conserved internal C-box and C-terminal IR (isoleucine-arginine) tail are boxed. *, identity; :, high similarity; ., some similarity.

We immunoprecipitated MYC-CORT from ovary extracts using a MYC antibody. Cdc27 and Cdc16, tetratricopeptide repeat core subunits of the APC/C, co-immunoprecipitate with MYC-CORT but not with a control mouse immunoglobulin G (IgG) (Figure 1A) (unpublished data). Morula (MR), the APC2 homolog in Drosophila, does not co-immunoprecipitate with MYC-CORT (Figure 1A). Data from S. cerevisiae suggest that the APC/C exists in two distinct subcomplexes bridged together by Apc1. One subcomplex contains Apc2 and Apc11, while the other contains the tetratricopeptide proteins Cdc27, Cdc16, and Cdc23 [45]. Failure of MR to co-immunoprecipitate with MYC-CORT may be explained by a tighter and more direct association of CORT with the tetratricopeptide protein-containing subcomplex but not with the Apc2-containing subcomplex. Furthermore, buffer conditions may be causing CORT to dissociate from MR, as extensive high-salt washes of human APC cause dissociation of Apc2 and Apc11 from the rest of the complex [46]. On the basis of previous studies of APC/C, we still think it is likely that CORT acts together with MR and Cdc27 in one complex. As an additional control, we immunoprecipitated an unrelated MYC-tagged protein, PLU-MYC, from ovary extracts to confirm that the associations of Cdc27 and Cdc16 with CORT are specific. Neither Cdc27 nor Cdc16 co-immunoprecipitate with PLU-MYC, indicating that they are associating with CORT and not with the MYC tag (Figure 1A) (unpublished data).

In the reciprocal experiment, we immunoprecipitated Cdc27 from ovary extracts (Figure 1B). MYC-CORT co-immunoprecipitates with Cdc27 but not with a control rabbit IgG, suggesting that there is a strong physical interaction between CORT and Cdc27.

In addition to demonstrating the physical association between CORT and the APC/C, we identified motifs in the CORT protein sequence that have been shown to contribute to binding of APC/C activator proteins to the APC/C (Figure 1C). CORT contains an internal motif called the C-box that is important for binding to the APC/C and is conserved in Cdc20 and Cdh1 proteins throughout many species [47,48]. In addition, CORT contains the C-terminal IR (isoleucine-arginine) tail motif that is present in all APC/C activators as well as Doc1 and has been shown to mediate a direct interaction between Cdh1 and Cdc27 [46,49]. Together, the co-immunoprecipitation of CORT with core subunits of the APC/C in oogenesis and the presence of conserved motifs in the CORT sequence confirm CORT's identity as a meiosis-specific APC/C activator.

### *cortex* Cannot Provide *fizzy* Function in the Early Embryo

An additional way to determine if *cort* functions as an APC/C activator is to ask whether it can provide the function of another APC/C activator when over- or misexpressed. To investigate whether *cort* acts similarly to *fzy*, we asked if *cort* can functionally substitute for *fzy* in the early embryo. *fzy* ^6^/*fzy* ^7^ females lay eggs that arrest in metaphase after a few rounds of mitotic divisions [50]. If *cort* can provide *fzy* function, we would expect these embryos to progress further in embryogenesis.

We overexpressed *myc*-*cort* in the germline of *fzy* ^6^/*fzy* ^7^ females, collected embryos from these females, visualized the DNA and the spindles by immunofluorescence, and counted the number of mitotic spindles. Embryos overexpressing *cort* did not contain more mitotic spindles compared to *fzy* alone, thus we did not observe any rescue of the *fzy* phenotype. In contrast, overexpression of *cort* slightly worsened the *fzy* phenotype (presented below). This result suggests either that *cort* does not function as an APC/C activator, or, more likely, that CORT confers significantly different substrate specificity to the APC/C than FZY and, therefore, cannot provide its function.

### *cortex* Triggers Degradation of Cyclin A Early in Meiosis I and Additional Substrates Later

In mitosis, cyclins are degraded sequentially by the APC/C, with Cyclin A being degraded prior to Cyclin B and Cyclin B3 [51,52]. For a more detailed analysis of CORT function in meiosis, we examined levels of putative APC/C^CORT^ substrates at different time points during the meiotic divisions. We can isolate different meiotic stages by dissecting egg chambers from ovaries: immature ovaries with egg chamber stages 1–12 contain oocytes arrested in prophase I, and mature stage 14 oocytes are arrested in metaphase I.

**Figure 2:**
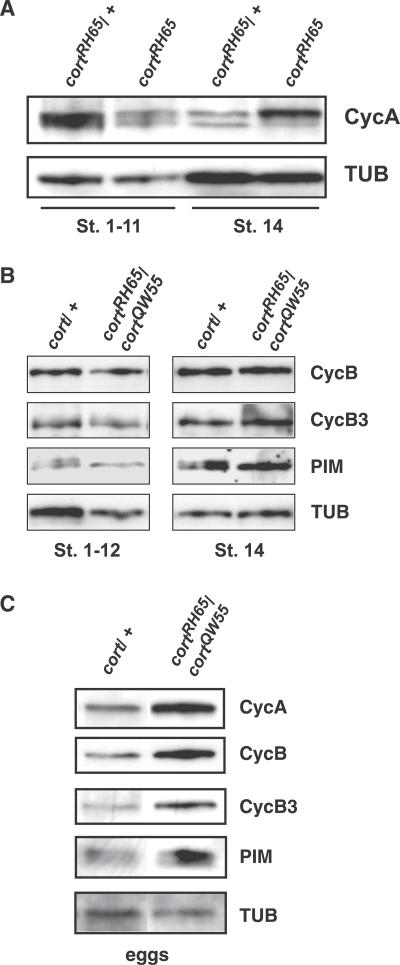
CORT Targets Sequential Degradation of APC/C Substrates in Meiosis (A) Cyclin A levels are elevated in *cort* mutant stage 14 oocytes. Western blot showing Cyclin A levels in immature stage 1–11 egg chambers and mature stage 14 oocytes from *cort^RH65^*/*+* or *cort^RH65^* females. The two forms of Cyclin A protein differ by phosphorylation state (L. Vardy, J.A. Pesin, and T.L. Orr-Weaver, unpublished data). The band intensities for each of the proteins were compared as ratios to the signal from tubulin to control for protein loading. By ImageJ analysis, the band intensity of Cyclin A in *cort^RH65^/+* stage 1–11 egg chambers is approximately 1.25-fold greater than Cyclin A in *cort^RH65^* stage 1–11 egg chambers. The band intensity of the more slowly migrating form of Cyclin A in *cort^RH65^* stage 14 oocytes is approximately 2.1-fold greater than the same Cyclin A band in *cort^RH65^/+* stage 14 oocytes. When assessing changes between developmental stages, Cyclin A band intensity in *cort^RH65^/+* stage 1–11 egg chambers is approximately 4.1-fold greater than *cort^RH65^/+* stage 14 oocytes. Cyclin A band intensity in *cort^RH65^* stage 1–11 egg chambers is approximately 2.4-fold greater than *cort^RH65^* stage 14 oocytes. Blot was probed with anti-α-Tubulin as a loading control. (B) Cyclin B, Cyclin B3, and PIM levels are unchanged in *cort* mutant ovaries. Western blot showing levels of Cyclin B, Cyclin B3, and PIM/Securin in immature stage 1–12 egg chambers and mature stage 14 oocytes from *cort^RH65^*/+ or *cort^RH65^*/*cort^QW55^* females. A doublet of PIM protein has been noted previously in some strain backgrounds [86]. Blot was probed with anti-α-tubulin as a loading control. All lanes are from the same blot. (C) Cyclin A, Cyclin B, Cyclin B3, and PIM/Securin levels are elevated in eggs laid by *cort^RH65^*/*cort^QW55^* females. By ImageJ analysis, band intensities are greater by the following approximate folds in *cort^RH65^*/*cort^QW55^* compared to heterozygous controls: Cyclin A, 1.7; Cyclin B, 2.4; Cyclin B3, 2.3; and PIM, 1.7. Western blot was probed with anti-α-Tubulin as a loading control. Nonconsecutive lanes from the same blot are shown.

We performed western blots on extracts from immature egg chamber stages and stage 14 oocytes to assay levels of Cyclin A at these meiotic time points in *cort* mutants. We found that Cyclin A levels are slightly reduced in *cort* mutant immature ovaries (stages 1–11), although the significance of this effect is not clear as we have not observed any defects in these stages of *cort* mutant ovaries. Cyclin A levels are elevated in mutant stage 14 oocytes compared to a heterozygous control (Figure 2A). The heterozygous control blots indicate that Cyclin A is normally degraded at some point between release of the prophase I arrest and establishment of the metaphase I arrest. In *cort* mutants, the failure to degrade Cyclin A by the metaphase I arrest indicates that APC/C^CORT^ is required for Cyclin A degradation at this time. In contrast, levels of Cyclin B, Cyclin B3, and PIMPLES (PIM), the securin homolog in Drosophila, are not elevated in *cort* mutant stage 14 oocytes compared to heterozygous controls, suggesting that these proteins are not subject to degradation by APC/C^CORT^ at this developmental stage (Figure 2B).

Upon egg activation in Drosophila, the metaphase I arrest is released, and meiosis is rapidly completed as the egg is ovulated and laid [53]. Meiosis is completed regardless of whether the oocyte is fertilized. Thus, unfertilized eggs represent a time point just after the completion of meiosis. We examined cyclin levels in eggs laid by *cort* mutant females, which do not complete meiosis (Figure 2C). As a control we used heterozygous unfertilized eggs. We found that all three mitotic cyclins, as well as PIM, are elevated in *cort* mutant eggs, which suggests that APC/C^CORT^ targets all of these substrates for degradation after release of the metaphase I arrest. Complementary results for cyclin levels have been previously observed [30]. This degradation may take place at the metaphase I to anaphase I transition, the metaphase II to anaphase II transition, or during both transitions. Cyclin B, at least, is likely degraded at both transitions as expression of a nondegradable form of Cyclin B in the female germline results in both meiosis I and meiosis II arrests [30].

The sequential CORT-dependent degradation of cyclins we observe in *Drosophila* female meiosis is parallel to observations made in mitosis that degradation of Cyclin A begins just after nuclear envelope breakdown in prometaphase, while degradation of Cyclin B is initiated at the beginning of metaphase [52,54–56]. Nuclear envelope breakdown occurs in Drosophila female meiosis in stage 13, and just after this stage is when we see an increase of Cyclin A but not Cyclins B or B3 in *cort* mutants compared to heterozygous controls. We see CORT-dependent degradation of all three cyclins in eggs, consistent with degradation of Cyclin B and Cyclin B3 not occurring until the metaphase I to anaphase I transition. To our knowledge, this is the first observation of sequential cyclin degradation during meiosis in a metazoan.

### Developmental Regulation of Appearance of CORT Protein in Female Meiosis

Given the difference in timing of CORT-dependent degradation of cyclins, we examined the protein expression pattern of CORT during meiosis to see if differential protein regulation of CORT correlates with differential cyclin degradation. Eggs laid by *cort* mutant mothers arrest in metaphase II and never complete meiosis [44]. This strong arrest phenotype indicates a critical role for *cort* specifically in meiosis. However, *cort* mRNA is present throughout oogenesis and early embryogenesis, suggesting a much broader developmental role [42]. We determined the timing of CORT protein expression to define better the scope of its activity.

**Figure 3:**
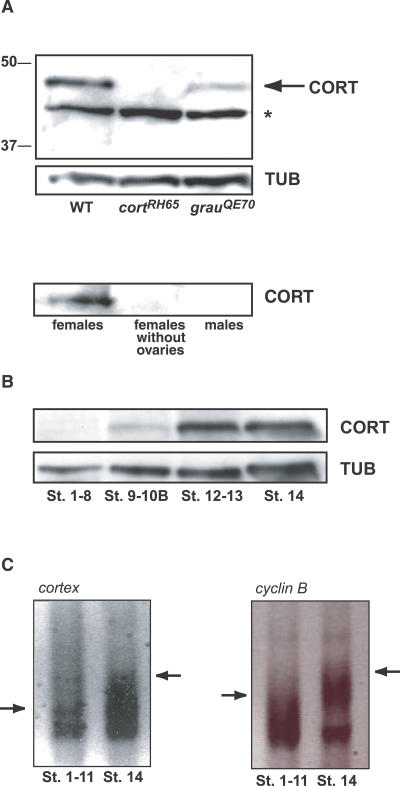
The Appearance of CORT Protein in Mature Oocytes Correlates with Polyadenylation of *cort* mRNA (A) Top panel: Characterization of CORT antibody. Western blot showing that anti-CORT serum recognizes a band of approximately 47 kDa in wild-type oocyte extracts. The band is absent in *cort^RH65^* mutant oocyte extracts and reduced in *grau^QE70^* mutant oocyte extracts. The blot was probed with anti-α-Tubulin as a loading control. Asterisk marks a nonspecific protein that reacts with anti-CORT serum. Size markers in kilodaltons are shown on left side of blot. Bottom panel: CORT expression is specific to oogenesis. Western blot shows presence of CORT in whole female fly extracts but absence from whole female extracts from which the ovaries were removed or from whole male extracts. (B) Developmental western blot of CORT in developing egg chambers. Endogenous CORT levels are low to undetectable in immature ovaries (stages 1–10B), but strongly increase in mature oocytes (stages 13–14). Extracts were made from hand-dissected ovaries. By ImageJ analysis, band intensity of CORT in stages 12–13 is 3.6-fold greater and in stage 14 3.5-fold greater than that of CORT in stage 9–10B. The blot was probed with anti-α-Tubulin as a loading control. (C) PAT assays showing elongation of poly(A) tail lengths of *cortex* and *cyclin B* mRNA upon oocyte maturation in wild-type ovaries (transition from immature stage 1–11 egg chambers to mature stage 14 oocytes). Arrows indicate the top of each poly(A) smear.

To investigate the developmental control of CORT protein expression, we made a polyclonal antibody against a glutathione S-transferase (GST)-tagged N-terminal fragment of CORT. Anti-CORT serum recognizes a band of approximately 47 kDa in wild-type oocyte extracts (Figure 3A). To test for antibody specificity, we probed oocyte extracts from *cort^RH65^* mutants that contain a *cort* allele with a premature stop codon [42]. The serum does not recognize a band of the same size in these mutants. We also did not detect an N-terminal fragment in this mutant extract. In addition, we probed extracts from *grauzone* (*grau*) mutant oocytes. *grau* encodes a transcription factor that activates expression of *cort* [57]. The CORT band of 47 kDa is reduced in *grau* mutants, confirming the specificity of our antibody.

CORT expression is specific to oogenesis, as we detected a CORT band in whole female fly extracts but not in female fly extracts from which the ovaries were removed (Figure 3A). We also did not detect CORT in whole male fly extracts. We performed developmental western analysis on different stages of oogenesis to determine specifically when CORT protein is expressed (Figure 3B). CORT is undetectable in early stage 1–8 egg chambers, and very low levels are detectable in stages 9–10B egg chambers. CORT levels increase dramatically in stage 12–13 egg chambers and remain high in mature stage 14 oocytes. The appearance of CORT protein occurs at the same time that Cyclin A degradation is triggered (Figure 2A), indicating that APC/C^CORT^ targets Cyclin A as soon as CORT protein is expressed, while simultaneously being prevented from targeting Cyclins B and B3 and PIM until after release of the metaphase I arrest.

The timing of appearance of CORT protein correlates with timing of the unmasking of maternal mRNA by cytoplasmic polyadenylation. Many organisms utilize cytoplasmic polyadenylation as a strategy to turn on the translation of specific transcripts at specific developmental time points [58]. Elongation of the poly(A) tail of these transcripts is thought to allow for a stable closed-loop conformation of the translational machinery and thus to activate translation. This process occurs during oocyte maturation when oocytes are released from prophase I arrest to reenter the meiotic cell cycle [2]. In *Drosophila*, oocyte maturation takes place in stage 13 of oogenesis [59]. Given the correlation of the appearance of CORT protein with the timing of oocyte maturation, we investigated the lengths of *cort* poly(A) tails at different developmental time points.

We conducted ligase-mediated poly(A) tail (PAT) assays on immature egg chambers and mature stage 14 oocytes to determine if the poly(A) tail length of *cort* changes upon oocyte maturation [60]. We observed an elongation of the poly(A) tail in stage 14 oocytes compared with stage 1–11 egg chambers (Figure 3C). Poly(A) tails are approximately 20 As in immature stages and elongate to approximately 70 As in mature oocytes. As a positive control, we measured the poly(A) tail length of *cyclin B* mRNA in these stages, because *cyclin B* mRNA is known to be polyadenylated upon oocyte maturation (Figure 3C) [61]. We observed a similar increase in *cyclin B* mRNA poly(A) tail lengths as has been previously shown. The appearance of CORT protein in stage 13 of oogenesis when oocyte maturation occurs together with the elongation of *cort*'s poly(A) tail in mature oocytes suggests that *cort* translation is developmentally regulated by cytoplasmic polyadenylation.

### *cort* mRNA Becomes Deadenylated in Early Embryogenesis

If APC/C^CORT^ activity is necessary for meiosis but dispensable for mitosis, *cort* may be inactivated in the early embryo to prevent its association with a mitotic APC/C complex. Early *Drosophila* embryos are transcriptionally quiescent, so post-transcriptional control is essential for regulating the activity of maternal gene products. In many organisms, egg activation triggers destabilization of a subset of maternal transcripts [58]. As deadenylation is often the rate-limiting step in mRNA decay, we investigated the polyadenylation status of *cort* mRNA after the completion of meiosis.

**Figure 4:**
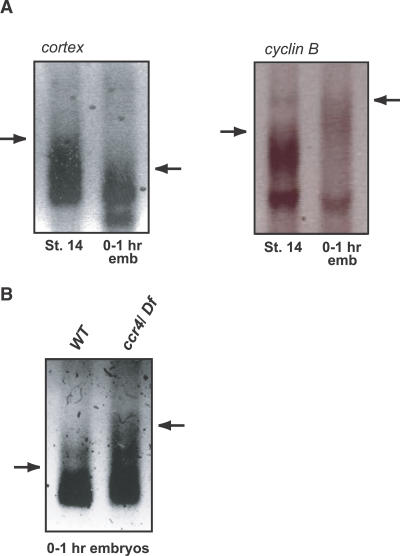
*cort* mRNA Is Deadenylated in Early Embryos (A) PAT assays showing shortening of poly(A) tail length of *cortex* mRNA and elongation of poly(A) tail length of *cyclin B* mRNA in wild-type stage 14 oocytes and 0–1-h embryos. Arrows indicate the top of each poly(A) smear. (B) Deadenylation of *cort* mRNA requires *ccr4*. PAT assay showing elongation of poly(A) tail length of *cortex* mRNA in 0–1-h embryos from *ccr4*/*Df* mutant mothers compared to wild-type control embryos. Arrows indicate the top of each poly(A) smear.

We performed PAT assays to measure the poly(A) tail length of *cort* mRNA in mature stage 14 oocytes and 0–1-h embryos. We found that *cort* mRNA is deadenylated in early embryos compared to mature oocytes (Figure 4A). The tail decreases from a length of approximately 70 As to 20 As. We used *cyclin B* mRNA as a positive control that, in contrast, is further polyadenylated upon egg activation [61]. CCR4 is the main catalytic subunit of the Ccr4-Pop2-Not deadenylase complex in S. cerevisiae [62]. A CCR4 homolog exists in *Drosophila* and has deadenylase activity [63]. We measured the poly(A) tail length of *cort* mRNA in embryos from *ccr4* mutant mothers and found that *cort*'s poly(A) tail length is elongated in the mutant (Figure 4B). Thus, deadenylation of *cort* in the early embryo is dependent on the conserved CCR4-NOT deadenylase complex.

### CORT Protein Becomes a Target of the APC/C after the Completion of Meiosis

**Figure 5:**
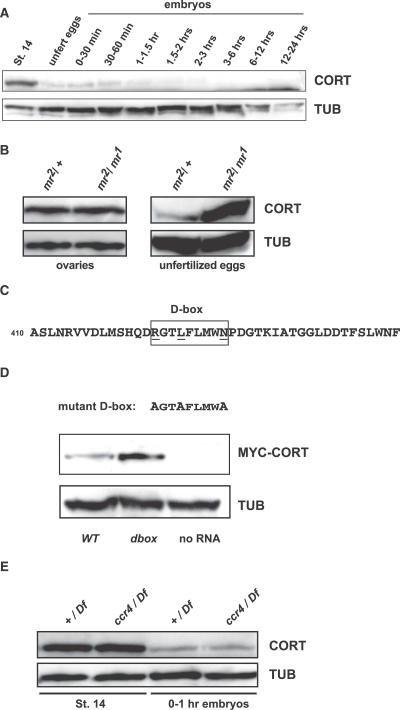
CORT Protein Becomes a Target of the APC/C after the Completion of Meiosis (A) Developmental western blot showing rapid drop in CORT levels in wild-type unfertilized eggs compared to stage 14 oocytes. Staged embryo extracts show decreasing levels of CORT as embryogenesis proceeds. Blot was probed with anti-α-Tubulin as a control. (B) CORT levels increase in *mr* mutants after the completion of meiosis. Western blot showing CORT levels in *mr^2^*/+ and *mr^2^*/*mr^1^* ovaries and unfertilized eggs. By ImageJ analysis, band intensity of CORT in *mr^2^*/+ and *mr^2^*/*mr^1^* ovaries is approximately identical. Band intensity of CORT in *mr^2^*/*mr^1^* eggs is approximately 3.4-fold greater than CORT in *mr^2^*/+ eggs. Blots were probed with anti-α-Tubulin as a loading control. (C) Putative D-box sequence in C terminus of CORT (residues 424–432). Conserved residues are underlined. (D) D-box sequence in CORT is required for its degradation in early embryos. All three conserved residues were mutated to alanine in D-box mutant CORT. Embryos (0–30 minute) were injected with *myc-cort* wild-type (*WT*) or D-box mutant (*dbox*) RNA or buffer alone (no RNA). Western blot showing levels of wild-type or D-box mutant MYC-CORT in injected embryos. D-box mutant CORT is stabilized compared to wild-type CORT. By ImageJ analysis, band intensity of D-box mutant MYC-CORT is approximately 2.7-fold greater than wild-type MYC-CORT. This blot is representative of five independent experiments, which all gave similar results. Blot was probed with anti-α-Tubulin as a control. (E) *ccr4* is not required to lower CORT protein levels in early embryos. Western blot showing levels of CORT in *ccr4*/*Df* and *+*/*Df* sibling control stage 14 oocytes and 0–1-h embryos. Blots were probed with anti-α-Tubulin as a loading control.

We performed western analysis on CORT after the completion of meiosis to determine when CORT protein is expressed in the early embryo (Figure 5A). Surprisingly, we found that CORT protein levels drop dramatically by the time meiosis is completed in unfertilized eggs. We detect CORT at very low levels for up to 1.5 h of embryogenesis before it disappears. Given the rapid timing of CORT degradation by the end of the meiotic divisions, we hypothesized that CORT itself may be a target of the APC/C.

To test whether the APC/C plays a role in CORT degradation, we looked at CORT levels in *mr/APC2* mutants (Figure 5B). We found that CORT levels are unaffected in *mr* mutant ovaries, but CORT levels increase strongly in eggs from *mr* mutant females. As a positive control, we probed for Cyclin B in these samples and found that it is also elevated in *mr* mutant eggs (unpublished data). These results parallel the timing of changes in CORT protein levels in a wild-type background; CORT levels normally drop by the time that meiosis is completed, and, similarly, the dependence of CORT degradation on *mr* is only apparent in unfertilized eggs, in which meiosis has been completed. These results strongly suggest that CORT is targeted for degradation by the APC/C at some point after the release of the metaphase I arrest and by the time that the meiotic divisions are completed. The specific timing of CORT degradation suggests that it is critical for development of the embryo that CORT protein levels be greatly reduced by the time meiosis is completed.

The APC/C targets proteins for degradation through recognition of specific motifs in its substrates. The two most common motifs are D-boxes (R-X-X-L-X-X-X-X-N) and KEN boxes (K-E-N-X-X-X-E/D/N), although additional motifs have been identified [4,14,64]. We identified a putative D-box in the C terminus of CORT (residues 424–432) but no KEN box (Figure 5C).

To determine whether the putative D-box in CORT is functional, we constructed a D-box mutant form and asked whether protein stability is affected in an embryo injection experiment. We mutated all of the conserved residues in CORT's D-box to alanine (Figure 5B) and tagged it with MYC to distinguish the protein from endogenous CORT. We know that a MYC-tagged form of CORT is regulated in a similar way to endogenous CORT, because transgenic MYC-tagged CORT is degraded with similar developmental timing in embryos to endogenous CORT in vivo (Figure S1). MYC-tagged D-box mutant *cort* and MYC-tagged wild-type *cort* were transcribed in vitro. The RNA was microinjected into 0–30-min post-deposition wild-type embryos. After incubating the embryos to allow for translation of the RNA and post-translational modifications of the proteins, extracts were made and analyzed by western blot (Figure 5D). We found that D-box mutant CORT is stabilized compared to wild-type CORT. Thus, the D-box motif in CORT is required for its degradation in early embryos.

Given our previous observation that *cort* mRNA is deadenylated in early embryos, we wondered if this regulatory mechanism also contributes to the drop in CORT protein levels after the completion of meiosis. To determine whether Ccr4-mediated deadenylation of *cort* mRNA is required for low levels of CORT in early embryos, we looked at CORT protein levels in *ccr4* mutants by western blot (Figure 5E). We found that CORT protein levels are unchanged in both *ccr4/Df* stage 14 oocytes and 0–1-h embryos when compared to heterozygous sibling controls. This result suggests that although *ccr4* is required for *cort* deadenylation, it is not required for a subsequent decrease in protein levels. Thus, APC/C-mediated degradation of CORT is the primary mechanism by which CORT protein levels are lowered in early embryos. It is likely that deadenylation serves as a backup mechanism to block future synthesis of CORT after fertilization.

### CORT Is Likely to Be Targeted for Degradation by APC/C^FZY^

Given the dependence of CORT degradation on *mr* and an intact D-box, we wanted to determine which APC/C activator is responsible for CORT's destruction. FZR protein is undetectable in 0–2-h embryos and, thus, is not a good candidate [65]. FZY, however, is present in 0–2-h embryos and is the most likely activator of APC/C-mediated degradation of CORT [65].

To test this hypothesis, we first looked at CORT levels in eggs laid by *fzy* mutant females. We were unable to detect an increase of CORT protein in these embryos, but these alleles are hypomorphic and may not show an effect on CORT (unpublished data). Next, we looked for genetic interactions between *cort* and *fzy*. If CORT is a substrate of APC/C^FZY^ in the early embryo, we would expect them to antagonize each other in a genetic pathway. Reducing the level of *cort* expression in *fzy* mutants should suppress the *fzy* phenotype, and increasing the amount of *cort* expression in *fzy* mutants should enhance the *fzy* phenotype.

**Figure 6:**
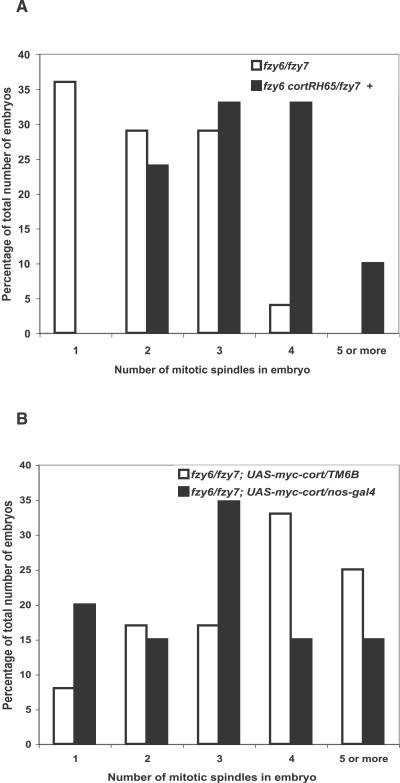
*cort* and *fzy* Genetically Interact in Early Embryos (A) *cort* dominantly suppresses the *fzy* female-sterile phenotype. Bar graph showing the percentage of 0–3-h embryos arresting with one, two, three, four, and five or more mitotic spindles. For *fzy^6^/fzy^7^*, *n* = 28. For *fzy^6^ cort^RH65^/fzy^7^ +*, *n* = 21. Analysis of the data with the Exact Wilcoxon Rank Sum Test generates a *p*-value of 3.43 × 10^−5^ when comparing the difference in the median number of mitotic spindles per embryo of *fzy^6^/fzy^7^* and *fzy^6^ cort^RH65^/fzy*. (B) Overexpression of *cort* in the female germline enhances the *fzy* female-sterile phenotype. Bar graph showing the percentage of 2–4-h embryos arresting with one, two, three, four, and five or more mitotic spindles. For *fzy^6^/fzy^7^; UAS-myc-cort/TM6B*, *n* = 12. For *fzy^6^/fzy^7^; UAS-myc-cort/nos-gal4*, *n* = 20. Analysis of the data with the Exact Wilcoxon Rank Sum Test generates a *p*-value of 0.12 when comparing the difference in the median number of mitotic spindles per embryo of *fzy^6^/fzy^7^; UAS-myc-cort/TM6B* and *fzy^6^/fzy^7^; UAS-myc-cort/nos-gal4*.

We carried out these genetic tests using *fzy* female-sterile mutants in which embryos arrest in metaphase after a few mitotic divisions [50]. Reducing the gene copy number of *cort* by one in a *fzy* mutant background causes a modest suppression of the *fzy* phenotype. Over 75% of embryos laid by these double mutant females arrest with three or more mitotic spindles, whereas only 33% of embryos from *fzy* single mutants develop this far (Figure 6A). Conversely, overexpressing *cort* in the germline of *fzy* mutant females slightly enhances the *fzy* phenotype. In this case, *fzy* embryos containing excess *cort* arrest with fewer mitotic spindles compared to *fzy* alone (Figure 6B). The results of these genetic interaction tests are consistent with CORT being a substrate of APC/C^FZY^ and suggest that the arrest phenotype of *fzy* embryos is due in part to the presence of excess CORT protein.

## Discussion

In this study, we investigated the function of *cort* and its developmental regulation in *Drosophila* female meiosis. We found that CORT protein physically associates with the APC/C in vivo and confirmed its function as an APC/C activator. We looked at levels of mitotic APC/C substrates in *cort* mutants and found that Cyclin A is targeted for destruction by APC/C^CORT^ in mature metaphase I arrested oocytes while Cyclin B, Cyclin B3, and PIMPLES/Securin are not targeted until egg activation. Developmental analysis of CORT protein showed that it is translated at oocyte maturation, and appearance of the protein correlates with polyadenylation of *cort* mRNA. Finally, we found that *cort* is regulated post-transcriptionally and post-translationally after the completion of meiosis. *cort* mRNA is deadenylated in early embryos, and CORT protein is degraded after egg activation in an APC/C-dependent manner. CORT contains a conserved D-box motif that is required for the efficiency of its degradation. Our results shed light on the mechanism for the regulation of a meiosis-specific APC/C activator, which results in restriction of its expression to a specific developmental time point.

### CORT Is an Activator of the APC/C

In this study, our demonstration of a physical interaction between CORT and the APC/C strengthens and confirms previous suggestions that *cort* encodes a functional meiosis-specific APC/C activator. A strong metaphase II arrest phenotype in *cort* mutant eggs and distant homology to the Cdc20/FZY protein family initially suggested that CORT might function as an APC/C activator [42,44]. More recently, *cort* was shown to negatively regulate levels of mitotic cyclin proteins, which is consistent with a role for CORT in activating the APC/C [30]. However, biochemical evidence linking CORT to the APC/C in vivo is crucial for this argument. We have shown that CORT physically associates with core subunits of the APC/C in ovaries, strongly supporting CORT's role as an APC/C activator.

### Developmental Control of CORT Levels

Coordination of the meiotic divisions with oogenesis and the transition from meiosis to restart of the mitotic cell cycle in embryogenesis present unique regulatory challenges for the organism. Our studies of *cortex* in *Drosophila* suggest that developmental control of levels of a meiosis-specific APC/C activator is one way in which meiosis is developmentally regulated, which has not been previously observed in a multicellular organism. This strategy exploits ongoing regulatory mechanisms occurring during meiosis and embryogenesis: cytoplasmic polyadenylation during oocyte maturation, deadenylation after egg activation, and APC/C-dependent degradation in the early embryo.

Cytoplasmic polyadenylation upon oocyte maturation has been shown to translationally activate maternal transcripts of genes that are required for meiotic entry, transition between meiosis I and meiosis II, and metaphase II arrest in vertebrates [58]. We have shown that *cort* mRNA is polyadenylated at oocyte maturation, which adds an APC/C subunit to this group of transcripts that are translationally unmasked for entry into the meiotic divisions. What is the signal for polyadenylation of *cort*? Masked transcripts contain a *cis*-acting cytoplasmic polyadenylation element (CPE) to which CPE binding protein (CPEB) is bound. Phosphorylation of CPEB upon oocyte maturation triggers elongation of the poly(A) tail and activation of translation [66]. We have not yet identified a CPE in the 3′ UTR of *cort*, although CPE sequences are quite variable. In addition, we have tested but have not observed a dependence of *cort* polyadenylation on *orb*, the CPEB homolog in *Drosophila* (unpublished data). Because the *orb* alleles we used are hypomorphic, we cannot fully rule out the possibility that polyadenylation of *cort* is *orb*/CPEB-dependent.

Egg activation triggers maternal transcript destabilization in several organisms, some of which occurs through *ccr4*-dependent deadenylation, and this is likely to be important for localization of maternal transcripts in the embryo and proper zygotic development [58]. We showed in this study that *cort* mRNA is deadenylated in the early embryo in a *ccr4*-dependent manner, but this deadenylation is not required for lowering CORT protein levels. However, we may not be able to detect a difference in protein levels because of the rapid APC/C-dependent degradation of CORT protein that occurs after release of the metaphase I arrest. Deadenylation could serve as a backup mechanism to ensure that CORT protein levels remain low in the early embryo by destabilizing *cort* mRNA.

The APC/C drives degradation of Cyclin B and other substrates during the rapid syncytial mitotic divisions of early embryogenesis in *Drosophila* [50,65,67,68]. We found that CORT is targeted for APC/C-dependent degradation by the completion of meiosis in the early embryo. The targeting of an APC/C activator for degradation by another form of APC/C is not unprecedented, as APC/C^Cdh1^ targets Cdc20 for degradation in G1 [16,17,19].

Our data support the conclusion that CORT is targeted by APC/C^FZY^. First, FZY is thought to be the only activator present in early embryos [65]. Second, we show here that *cort* and *fzy* interact genetically in a way that is consistent with *cort* being a negative downstream target of *fzy* in embryos. Third, in our embryo injection experiments, we showed that exogenous MYC-CORT is degraded in a D-box–dependent manner in injected embryos. Because the only APC/C activator in early embryos is FZY, degradation of MYC-CORT is likely to occur through APC/C^FZY^ in this assay.

It is also possible the APC/C^CORT^ regulates itself in a negative feedback loop by targeting CORT for degradation when levels of CORT reach a certain threshold at the end of meiosis. To address this possibility, we looked at the degradation of CORT in a homozygous *cort^QW55^* background in which there is no functional CORT protein. CORT^QW55^ mutant protein is not degraded at the transition from mature stage 14 oocytes to unfertilized eggs, unlike in a heterozygous control background (unpublished data). These results suggest that CORT could be targeted by itself, but it remains a possibility that the lesion in the *cort^QW55^* allele prevents an interaction between CORT^QW55^ mutant protein and the APC/C machinery. The lesion does not disrupt the D-box, but it could affect proper folding and structure of the protein. In summary, we conclude that CORT is targeted for degradation by the APC/C. It is most likely that FZY is the participating APC/C activator, but CORT may also contribute to targeting itself for degradation.

### Role of Meiosis-Specific Activators in *Drosophila*

Recent work has shown that both *cort* and *fzy* are required for the meiotic divisions in *Drosophila* female meiosis. Mutant analysis suggests that *cort* and *fzy* act redundantly to control the metaphase I to anaphase I transition, whereas they seem to act with different temporal and spatial specificity in targeting Cyclin B for destruction along the meiosis II spindles [30]. We showed in this study that *cort* cannot functionally substitute for *fzy* in the early embryo, suggesting that they target nonredundant sets of substrates. However, in this experiment, we cannot rule out the possibility that MYC-CORT was not present in sufficient levels in early embryos for rescue because of low expression levels or protein instability (Figure S1). Although MYC-CORT is expressed at high levels in stage 14 oocytes, it appears to be subject to degradation after the completion of meiosis, like the endogenous CORT protein.

Furthermore, homozygous *cort* mutants alone exhibit a strong metaphase II arrest, indicating that the wild-type levels of *fzy* in this background are not able to act in place of *cort* to control passage through metaphase II [44]. Finally, we have observed that FZY is expressed at a uniform level during oogenesis and embryogenesis (Figure S2) (unpublished data), which is in contrast to our results in this study showing that CORT expression is specifically upregulated during the meiotic divisions. On the basis of all of these observations, we think it is likely that in addition to the mitotic cyclins, APC/C^CORT^ targets a unique set of substrates in meiosis that are not recognized by APC/C^FZY^. The identification of these meiotic substrates will be crucial for understanding how the meiotic divisions are controlled in the oocyte.

The study of meiotic control of the APC/C is especially intriguing in *Drosophila*, because in addition to *cort*, a female meiosis-specific activator, the genome contains *fizzy-related 2* (*fzr2*), another member of the Cdc20/FZY family. *fzr2* is expressed exclusively in testes and may act as a male meiosis-specific activator [69]. Further study of both *cort* and *fzr2* will be important for understanding differential developmental regulation of the APC/C during meiosis in females versus males.

### Sequential Degradation of Cyclins in Meiosis

In mitosis, cyclins are targeted sequentially for destruction by the APC/C. Degradation of Cyclin A begins just after nuclear envelope breakdown in prometaphase, while degradation of Cyclin B does not occur until the metaphase to anaphase transition [52,54–56]. Sequential degradation of Cyclin A, Cyclin B, and, finally, Cyclin B3 in *Drosophila* triggers a series of distinct events leading to exit from mitosis [51,70]. We have found that a similar situation exists in *Drosophila* female meiosis, in which degradation of Cyclin A by APC/C^CORT^ initiates upon nuclear envelope breakdown, but degradation of Cyclin B and Cyclin B3 does not occur until after the metaphase I to anaphase I transition.

The difference in timing of Cyclin A and Cyclin B degradation in mitosis is due to regulation of the APC/C by the spindle assembly checkpoint. The spindle assembly checkpoint inhibits APC/C^Cdc20^ from initiating anaphase until all chromosomes are bioriented on the spindle, in part through direct binding of Cdc20 to Mad2 and BubR1 [71]. Spindle assembly checkpoint proteins specifically inhibit APC/C-dependent ubiquitination of Cyclin B but not of Cyclin A [52,55,56]. APC/C^CORT^ may be regulated in a similar manner during meiosis I. Indeed, the spindle assembly checkpoint is likely to function during meiosis I in *Drosophila*, as the conserved spindle checkpoint kinase Mps1 is required for delaying entry into anaphase I to allow for proper segregation of achiasmate homologs and maintenance of chiasmate homolog connections in *Drosophila* oocytes [72,73]. Furthermore, a functional Mad2-dependent checkpoint exists during meiosis I in mouse oocytes, and spindle checkpoint components have been shown to regulate the APC/C during meiosis I in C. elegans [33,74,75].

To determine whether APC/C^CORT^ is regulated by the spindle checkpoint, we asked if BubR1 or Mad2 physically associate with CORT in stage 14-enriched ovaries. We were unable to detect an association with BubR1 or Mad2 (unpublished data). Although this negative result does not rule out the possibility of regulation of APC/C^CORT^ by the spindle checkpoint, it suggests that APC/C^CORT^ may be subject to other types of regulation that inhibit it from targeting Cyclin B and Cyclin B3 for degradation until after the metaphase I arrest.

In conclusion, through the investigation of *cortex*, a meiosis-specific APC/C activator, we have found one way in which the meiotic cell cycle may be developmentally controlled during oogenesis. *cort* is developmentally regulated by existing post-transcriptional and post-translational mechanisms, resulting in expression of CORT protein being restricted to the meiotic divisions. Further study of APC/C^CORT^ will continue to elucidate the ways in which developmental control of the APC/C contributes to proper female meiosis in a metazoan.

## Materials and Methods

### Fly stocks.

Crosses were performed, and flies were maintained between 22 °C and 25 °C using standard techniques [76]. The wild-type stocks used were *Oregon R* and *yw*. The *cort^RH65^* and *cort^QW55^* alleles have been described [42,44,77]. To obtain *ccr4* mutant flies, *ccr4^KG877^*, a *ccr4* allele generated by the Berkeley *Drosophila* Genome Project (http://www.fruitfly.org), was placed in *trans* to *Df(3R)crb-F89-*4, a large deficiency that deletes the *ccr4* locus [63]. Female-sterile alleles of *morula*, *mr^1^* and *mr^2^*, were originally isolated from natural populations and have been described [78–80]. Female-sterile alleles of *fizzy*, *fzy^6^* and *fzy^7^*, have been described [50]. UASp *myc-cort* was made by PCR amplification of *cort* cDNA (LD43270) and subcloning into pUASp with a 6xMYC tag at the N terminus. The LD43270 clone is missing coding sequence for nine amino acids on the 5′ end that we added during PCR amplification. Expression of *6xmyc-cort* was driven in the female germline with the *nanos-Gal4-VP16* driver [81]. The *plu-myc* transgenic line has been described [82]. To generate unfertilized eggs, we crossed virgin females to sterile males, which do not produce sperm but are able to stimulate females to lay eggs. The sterile males are from strain *T(Y;2)#11cn bw^D^ mr^2^/b cn mr^1^ bs^2^/SM6A*, a gift from B. Reed.

### Immunoprecipitations.

To prepare ovary extracts for immunoprecipitations, whole ovaries were dissected in Grace's insect medium (Gibco) from 32 females fattened 3 d on wet yeast at 25 °C. Ovaries were homogenized in 3× volume homogenization buffer (25 mM HEPES [pH 7.5], 0.4 M NaCl, 0.1 mM EDTA, 0.1 mM EGTA, 1 mM PMSF, 10% glycerol, complete mini EDTA-free protease inhibitors, 1 tablet/10 ml [Roche]), snap frozen in liquid nitrogen, and stored at −80 °C. A total of 30 μl Protein A Sepharose beads (Amersham) were precoupled to antibodies for 1 h at 4 °C. For precoupling, antibodies were as follows: 2 μl mouse IgG (Sigma I5381); 12 μl mouse monoclonal anti-myc, 9E10 (Covance); 2 μl rabbit IgG (Sigma I5006); or 10 μl affinity-purified rabbit anti-Cdc27 [68]. After removing an aliquot for input, 70 μl ovary extract was added to antibody-bound beads and incubated for 2–4 h at 4 °C. Beads were washed once in ice-cold IP buffer (25 mM HEPES [pH 7.5], 100 mM NaCl, 1 mM EGTA, 0.1% Triton X-100, 10% glycerol, complete mini EDTA-free protease inhibitors, 1 tablet/10 ml [Roche]), once in IP buffer plus 0.5 M NaCl, and 4 times in IP buffer. Inputs, immunocomplexes, and supernatants were resolved by SDS-PAGE and analyzed by immunoblot as described below.

### Polyclonal antibodies against CORT.

A fusion between GST and 152 amino acids from the N terminus of CORT was used to produce antibodies in guinea pigs. The construct encoding GST-CORT_N was made by PCR amplification of *cort* cDNA (clone number LD43270) as described above, followed by subcloning into pGEX-4T-1 expression vector (Pharmacia). GST-CORT_N was expressed in TOP10 E. coli cells by IPTG induction. The majority of GST-CORT_N was insoluble so it was gel purified from the insoluble material after cell lysis. Crude lysate was clarified, and the insoluble pellet resuspended in 5× Sample Buffer (60 mM 1 M Tris-HCl [pH 6.8], 25% glycerol, 2% SDS, 14.4 mM 2-mercaptoethanol, 0.1% bromophenol blue). Sample was resolved by SDS-PAGE on a preparative 10% 150:1 (30% acrylamide/2% bis-acrylamide) gel. Vertical strips from either side of the gel were stained with GelCode Blue (Pierce) and used as a guide to cut out the unstained band of GST-CORT_N. Gel slice was pulverized with cold 1× SDS Electrophoresis Buffer (25 mM Tris base, 192 mM glycine, 0.1% SDS) through a 10 ml syringe and gently rocked for 30 min at 4 °C to elute protein. Gel slice mixture was filtered through 125 μm nylon mesh (Tetko), and the eluate concentrated in Amicon Centricon YM-10 (Millipore). Concentrated protein was injected into guinea pigs for antibody production (Covance). The anti-CORT antibody recognizes a band of approximately 47 kDa that is the CORT protein.

### Protein extracts and immunoblotting.

Protein extracts were made by homogenizing staged egg chambers, whole ovaries, unfertilized eggs, or embryos in 3:1 Urea Sample Buffer (8 M urea, 2% SDS, 100 mM Tris [pH 7.5], 5% Ficoll)/embryo (vol/vol). Unfertilized eggs were collected for 0–2 h. Whole fly extracts were made by homogenizing flies directly in 5× Sample Buffer. Protein extracts were resolved by SDS-PAGE and transferred to Immobilon-P membranes (Millipore). We used 10.5%–14% acrylamide gels for immunoprecipitations (Figure 1) and substrate blots (Figure 2). We used 10% acrylamide gels for all CORT blots and RNA injection assays (Figures 3 and 5). Equal amounts of protein were loaded per lane and confirmed by anti-α-Tubulin blotting. Blots were probed with the following antibodies: mouse monoclonal anti-MYC, 9E10 (1:1000, Covance); affinity-purified rabbit anti-Cdc27 (1:500 [68]); affinity-purified rabbit anti-MR (1:200 [83]); guinea pig anti-CORT serum (1:2000); rat monoclonal anti-α-Tubulin, YL1/2 and YOL1/34 (1:200, Harlan Sera-lab); mouse monoclonal anti-Cyclin A, A19 (1:50, gift of P. O'Farrell); mouse monoclonal anti-Cyclin B, F2F4 (1:200 [84]); rabbit anti-Cyclin B3 serum (1:4000 [51]); rabbit anti-PIM serum (1:10,000 [85]); and affinity-purified rabbit anti-FZY (1:1000 [65]). Alkaline phosphatase- or horseradish peroxidase-conjugated secondary antibodies were used to detect bound primary antibodies. Protein was detected using ECL Plus (Amersham).

### PAT assays.

Ovary or embryo mRNA was isolated using the PolyATtract System 1000 (Promega). LM-PAT assays were performed using 100 ng mRNA as described [60]. cDNA was made using the Reverse Transcription System (Promega). PCRs were performed with message-specific primers, and a fraction of the PCR product was tested on a gel to permit approximately equal loading of the PCR product for the experiment. PCR products were separated on a 2% MetaPhore agarose gel and stained with ethidium bromide.

### Mutagenesis and RNA injection assays.

Point mutations were introduced into *cort* cDNA using the Phusion Site-Directed Mutagenesis Kit (Finnzymes). Wild-type or mutated D-box *cort* cDNA was subcloned into pCS2 containing a 6xMYC tag at the N terminus. Capped mRNAs were synthesized from these vectors using the SP6 mMessage mMachine Kit (Ambion). mRNA was purified using the MEGAclear Kit (Ambion). *yw* embryos that were 0–30 min postdeposition were dechorionated and prepared for injection. Samples were prepared containing 250 ng/μl wild-type or mutant *cort* RNA in injection buffer (5 mM KCl, 0.1 M K_2_HPO_4_ [pH 7.8]). A no RNA control contained injection buffer alone. Each sample was injected into 150 embryos. After 40 min at room temperature, the embryos were harvested in heptane, washed 2 times in embryo wash (0.4% NaCl, 0.03% Triton X-100), and homogenized in 20 μl USB. Extracts were resolved by SDS-PAGE and analyzed by immunoblotting as described above. The experiment was repeated 5 independent times to confirm results.

### Egg fixation, staining, and microscopy.

Eggs were collected for 0–3 h for Figure 6A and for 2–4 h for Figure 6B, dechorionated in 50% bleach, devitellinized in methanol and heptane, and fixed in methanol for 3 h at room temperature or overnight at 4 °C. Eggs were stained for DNA with Propidium Iodide and for Tubulin with rat monoclonal anti-α-Tubulin, YL 1/2, and YOL 1/34 (1:20, Harlan Sera-lab). Antibodies were detected using fluorescent secondary antibodies (Jackson Immunoresearch). Imaging was performed using a Zeiss Axioskop.

## Supporting Information

Figure S1 MYC-CORT Is Degraded in Early Embryos MYC-CORT expression was driven in the female germline with *nos-gal4* in a wild-type background. Western blot shows levels of MYC-CORT in stage 14 oocytes, unfertilized eggs, and developing embryos. MYC-CORT was detected by probing with anti-MYC. Control sibling females contain *nos-gal4* but not *UAS-myc-cort*. Blot was probed with anti-α-Tubulin as a loading control.
(2.2 MB PDF)

Figure S2 FZY Is Expressed at Uniform Levels during Embryogenesis Developmental western blot showing levels of FZY in stage 14 oocytes, unfertilized eggs, and developing embryos. Blot was probed with anti-α-Tubulin as a loading control.
(3.8 MB PDF)

Table S1 Rescue of *cort* Mutants by Tagged Transgenes (20 KB DOC)

### Accession Numbers

The FlyBase (http://flybase.bio.indiana.edu/search/) accession numbers for genes and gene products discussed in this paper are *bubR1* (FBgn0025458), *ccr4* (FBgn0011725), *cdc16* (FBgn0025781), *cdc27* (FBgn0012058), *cort* (FBgn0000351), *cycA* (FBgn0000404), *cycB* (FBgn0000405), *cycB3* (FBgn0015625), *fzr* (FBgn0003200), *fzr2* (FBgn0034937), *fzy* (FBgn0001086), *grau* (FBgn0001133), *mad2* (FBgn0035640), *mr* (FBgn0002791), *orb* (FBgn0004882), and *pim* (FBgn0003087).

## Abbreviations

APC/C: anaphase-promoting complex/cyclosome
CPE: cytoplasmic polyadenylation element
CPEB: CPE binding protein
*cort*: *cortex*
GST: glutathione S-transferase
IgG: immunoglobulin G
MR: morula
PAT assay: poly(A) tail assay
PIM: PIMPLES
