# Supplementary material for: Developmental Role and Regulation of cortex, a Meiosis-Specific Anaphase-Promoting Complex/Cyclosome Activator
Source: PLoS Genet. 2007 Nov 16;3(11):e202. doi: 10.1371/journal.pgen.0030202 (PMC2077894; doi:10.1371/journal.pgen.0030202)
Supplement: Supplementary file 1 [file pgen.0030202.sg001.pdf]

Pesin_FigS1

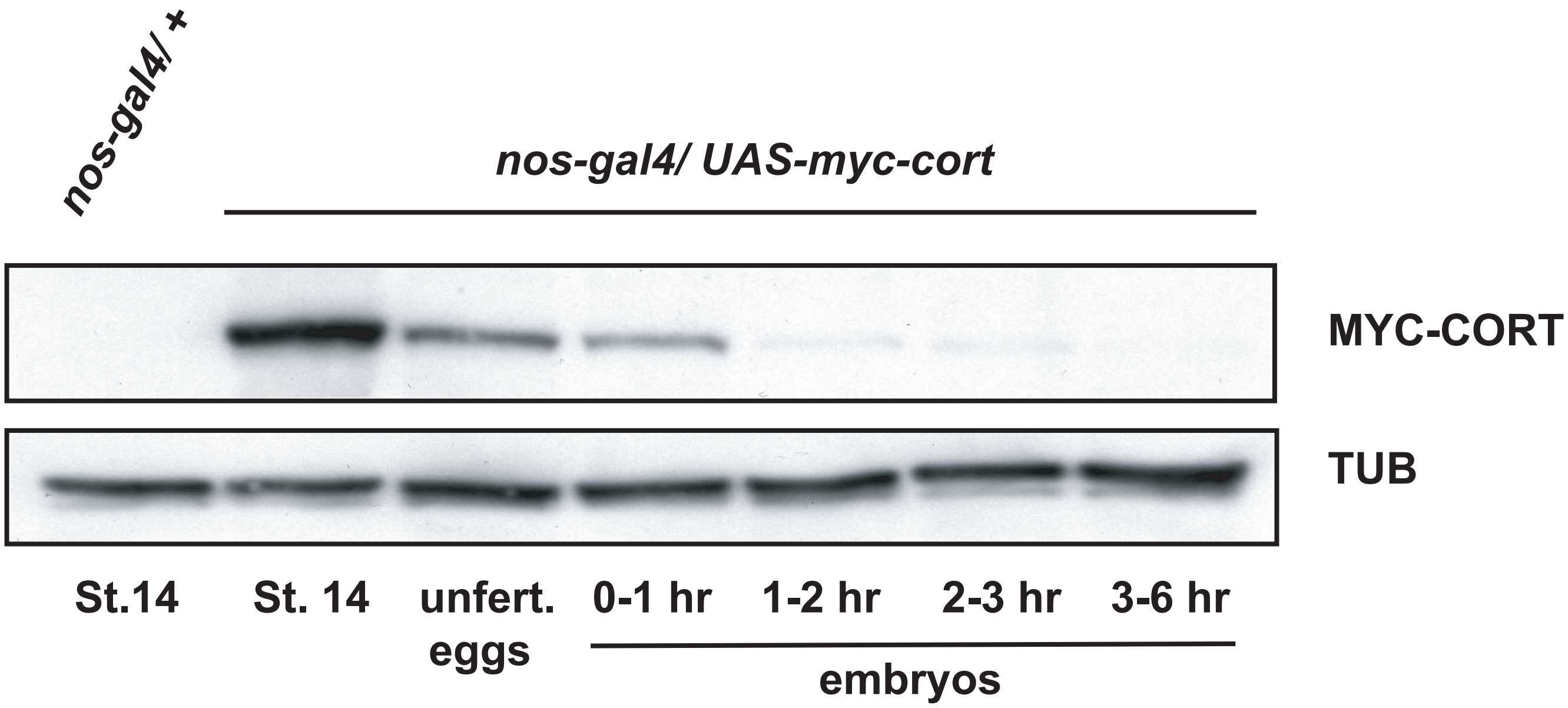
nos-gal4/ +
nos-gal4/ UAS-myc-cort
MYC-CORT
TUB
St.14
St. 14
unfert. eggs
0-1 hr
1-2 hr
2-3 hr
3-6 hr
embryos
