# Supplementary material for: Developmental Role and Regulation of cortex, a Meiosis-Specific Anaphase-Promoting Complex/Cyclosome Activator
Source: PLoS Genet. 2007 Nov 16;3(11):e202. doi: 10.1371/journal.pgen.0030202 (PMC2077894; doi:10.1371/journal.pgen.0030202)
Supplement: Supplementary file 2 [file pgen.0030202.sg002.pdf]

Pesin_FigS2

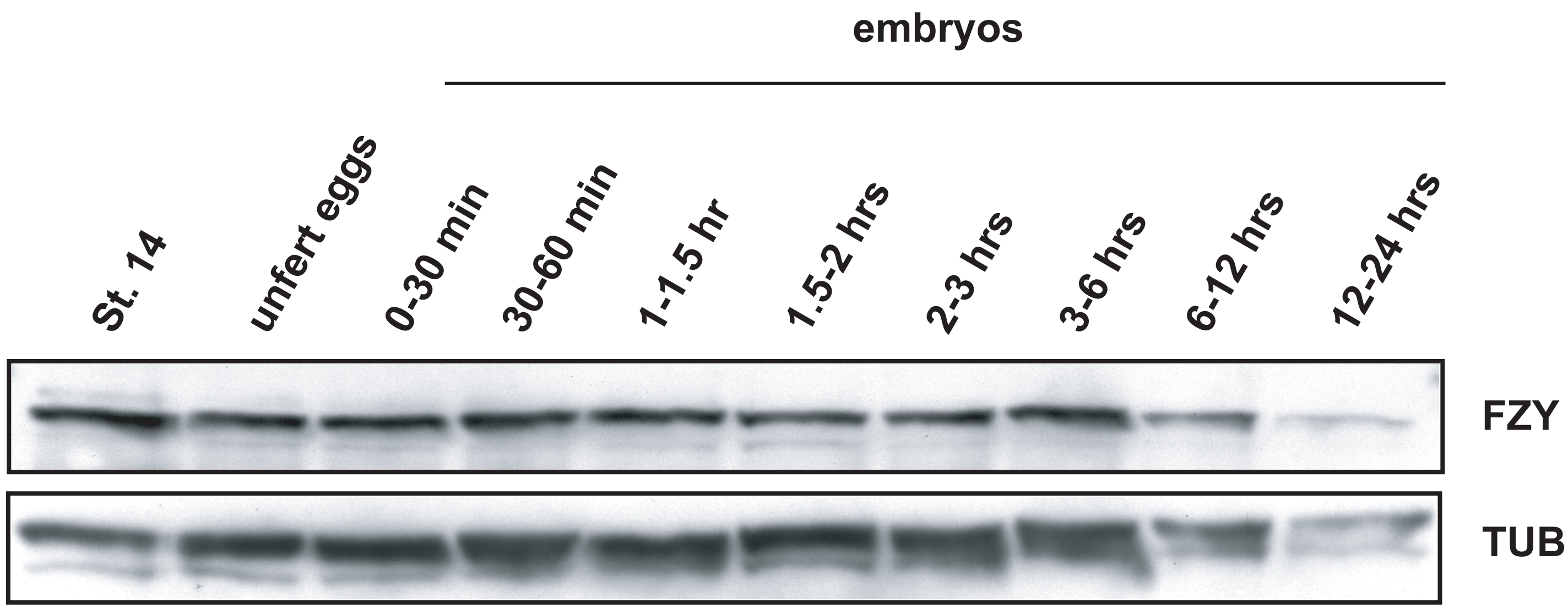
embryos
St. 14
unfert eggs
0-30 min
30-60 min
1-1.5 hr
1.5-2 hrs
2-3 hrs
3-6 hrs
6-12 hrs
12-24 hrs
FZY
TUB
