# Supplementary material for: Developmental Role and Regulation of cortex, a Meiosis-Specific Anaphase-Promoting Complex/Cyclosome Activator
Source: PLoS Genet. 2007 Nov 16;3(11):e202. doi: 10.1371/journal.pgen.0030202 (PMC2077894; doi:10.1371/journal.pgen.0030202)
Supplement: Supplementary file 3 [file pgen.0030202.st001.doc]

**Supplemental Table.** *Rescue of cort mutants by tagged transgenes*

Percentage of mitotically

Genotype	dividing embryos (*n*)

*cort^RH65^*/ *cort^RH65^*; *UAS-myc-cort^3C^*/ *TM6B*	0 (34)

*cort^RH65^*/ *cort^RH65^*; *UAS-myc-cort^3C^*/ *nos-gal4*	64 (44)

*cort^RH65^*/ *cort^RH65^*; *UAS-myc-cort^3A^*/ *nos-gal4*	26 (211)

Females of the indicated genotypes were fattened on wet yeast for 2 days before embryo collection. 2-4 hour embryos were dechorionated, methanol fixed, and stained with DAPI. Two independent transgenic lines are shown.
